# Establishing a *Klebsiella pneumoniae*-Based Cell-Free Protein Synthesis System

**DOI:** 10.3390/molecules27154684

**Published:** 2022-07-22

**Authors:** Chen Yang, Miaomiao Yang, Wanhua Zhao, Yue Ding, Yu Wang, Jian Li

**Affiliations:** 1 School of Physical Science and Technology, ShanghaiTech University, Shanghai 201210, China; yangchen1@shanghaitech.edu.cn; 2 Clinical Pathology Center, The Fourth Affiliated Hospital of Anhui Medical University, Hefei 230012, China; yangmiaomiaom@163.com; 3 Department of Biological Physics, University of Science and Technology of China, Hefei 230026, China; 4 College of Life Sciences, Jiangxi Agricultural University, Nanchang 330045, China; zwh17506998839@163.com (W.Z.); dyueyue0712@163.com (Y.D.)

**Keywords:** *Klebsiella pneumoniae*, cell-free protein synthesis, genome editing, cell-free synthetic biology

## Abstract

Cell-free protein synthesis (CFPS) systems are emerging as powerful platforms for in vitro protein production, which leads to the development of new CFPS systems for different applications. To expand the current CFPS toolkit, here we develop a novel CFPS system derived from a chassis microorganism *Klebsiella pneumoniae*, an important industrial host for heterologous protein expression and the production of many useful chemicals. First, we engineered the *K. pneumoniae* strain by deleting a capsule formation-associated *wzy* gene. This capsule-deficient strain enabled easy collection of the cell biomass for preparing cell extracts. Then, we optimized the procedure of cell extract preparation and the reaction conditions for CFPS. Finally, the optimized CFPS system was able to synthesize a reporter protein (superfolder green fluorescent protein, sfGFP) with a maximum yield of 253 ± 15.79 μg/mL. Looking forward, our *K. pneumoniae*-based CFPS system will not only expand the toolkit for protein synthesis, but also provide a new platform for constructing in vitro metabolic pathways for the synthesis of high-value chemicals.

## 1. Introduction

Cell-free protein synthesis (CFPS) systems, which use crude cellular extracts instead of intact cells to complete in vitro transcription and translation, are robust platforms for protein synthesis and biological applications [1,2,3]. Because of the open nature of cell-free systems, CFPS reactions have many advantages such as easy manipulation, high productivity, and tolerance of toxic products [4,5,6,7,8]. Using the CFPS technology, various proteins have been produced, including membrane proteins, therapeutic proteins, metalloenzymes, and unnatural amino acid-modified proteins [9,10,11,12]. Overall, recent efforts have constructed robust, cost-effective, and high-yield CFPS platforms for the rapid synthesis, study, and engineering of proteins.

CFPS systems have been developed from different prokaryotic and eukaryotic organisms such as *Escherichia coli*, yeast, wheat germ, and mammalian cells [13,14]. While these well-developed CFPS systems are widely used, each of them has its own advantages and drawbacks. For example, the overall productivity of the *E. coli* CFPS is currently the highest (~1000 μg/mL of expressed proteins) among all reported cell-free systems; however, *E. coli*-based cell extracts lack the post-translational modification machinery (e.g., glycosylation), and thus, they are not suitable for the expression of eukaryotic proteins. On the other hand, the preparation steps of eukaryotic organism-derived CFPS systems are laborious and their protein yields are often low (<50 μg/mL). Recently, the renewed interest in CFPS technology has motivated the development of new cell-free systems, which include *Streptomyces* species, *Bacillus subtilis*, *Pseudomonas putida*, and *Vibrio natriegens*, among others [15,16,17,18,19,20,21,22,23,24]. Developing new CFPS systems will not only expand the toolkit for in vitro protein synthesis, but also, more importantly, these systems can better mimic the cellular endogenous environment for high-quality protein expression (e.g., enhanced solubility and post-translational modification). In particular, the development of CFPS systems derived from non-model chassis microbes is becoming more attractive to defined applications in the field of synthetic biology and biotechnology.

**Figure 1:**
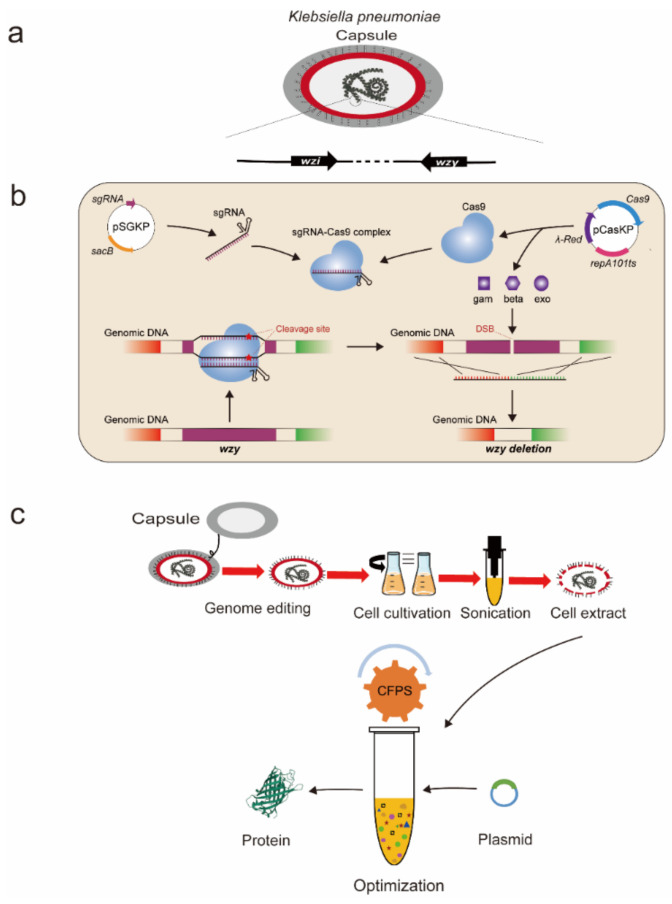
Development of a *K. pneumoniae*-based CFPS system. (**a**) The genes (*wzi* and *wzy*) from the capsule biosynthesis gene cluster in *K. pneumoniae*. (**b**) Deletion of the capsule-associated genes by a CRISPR-Cas9-based genetic tool using *wzy* as an example. (**c**) The process for preparing highly active cell extracts and optimization of the *K. pneumoniae* CFPS system for high-yield protein production.

*Klebsiella pneumoniae* strains are Gram-negative bacteria with an extracellular polysaccharide capsule produced by a capsule formation-associated gene cluster (Figure 1a). *K. pneumoniae* belongs to the *Enterobacteriaceae* family and distributes ubiquitously in the natural environment [25]. While *K. pneumoniae* is a human opportunistic pathogen in hospital infections [26], several *K. pneumoniae* strains have also been developed as efficient microbial cell factories for the production of valuable chemicals such as 2,3-butanediol, gluconic acid, 3-hydroxypropionic acid, and 1,3-propanediol [27,28,29,30]. In this work, we aim to develop a *K. pneumoniae*-based CFPS system, and thus will expand the current CFPS repertoire for more applications. To do this, we initially deleted the capsule-associated gene *wzy* to facilitate cell collection and lysis (Figure 1b). Then, we optimized the process of cell extract preparation and CFPS reaction conditions (Figure 1c). Under optimal conditions, the maximum yield of the reporter protein (superfolder green fluorescent protein, sfGFP) reached more than 250 μg/mL. To the best of our knowledge, this is the first report of using a *K. pneumoniae* strain to establish a high-yield CFPS system. Looking forward, we envision that our *K. pneumoniae* CFPS system together with various other efficient cell-free platforms, including the well-developed *E. coli* CFPS system, will provide broad utility for synthetic biology applications such as rapid protein synthesis, genetic circuit prototyping, and metabolic pathway construction for high-yield production of valuable chemicals and materials.

## 2. Results and Discussion

### 2.1. Genome Engineering of K. pneumoniae by Deleting Two Capsule-Associated Genes

Naturally, the wild-type *K. pneumoniae* KP_1.6366 strain is able to form a thick polysaccharide outer capsule, which makes capsulated cells more buoyant and hard to sediment after liquid cultivation [31]. Hence, the capsular polysaccharides might increase the broth viscosity of the culture and impede the downstream cell collection after cultivation. To tackle this problem, we initially attempted to construct non-capsulated *K. pneumoniae* mutants that can be used for easy cell biomass collection. To this end, we chose two capsule-associated genes, *wzi* and *wzy*, as our targets for disruption from the genome of KP_1.6366. The *wzi* gene encodes an outer membrane protein involved in capsular attachment to the cell’s surface, which is highly conserved in *K. pneumoniae* species [32]. The *wzy* gene has been predicted to encode a putative *O*-antigen polymerase from the capsular polysaccharide synthesis gene cluster of *K. pneumoniae* [33,34]. By deleting these two genes, we hope to disrupt the process of capsular polysaccharide biosynthesis and attachment to cells. Note that we just want to delete a large partial sequence (i.e., 54.7% and 65.4% of the gene length for *wzi* and *wzy*, respectively), but not the full sequence, of each gene in this work.

**Figure 2:**
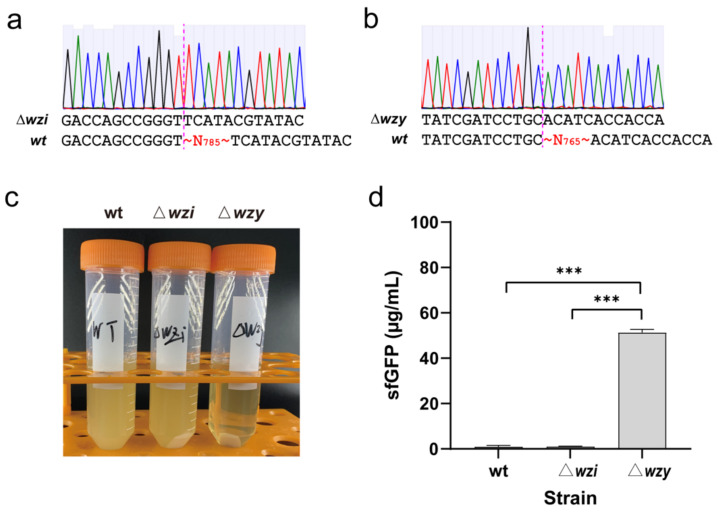
Deletion of the genes (**a**) *wzi* and (**b**) *wzy* from the genome of *K. pneumoniae* KP_1.6366. Both disrupted gene sequences were confirmed by DNA sequencing. (**c**) Cell collection of three strains by centrifugation at 12,000 g for 10 min. (**d**) Cell-free synthesis of sfGFP using cell extracts prepared from the wild-type (wt), Δ*wzi*, and Δ*wzy* strains. Values show means with error bars representing standard deviations (s.d.) of at least 3 independent experiments. Student’s *t*-tests were used for statistical analysis, and *p* < 0.05 indicated statistical significance (*** *p* < 0.001).

A previously developed CRISPR-Cas9/lambda-Red genetic tool was used to delete *wzi* and *wzy* [35]. The results indicated that partial sequences of both genes were successfully deleted from the genome, respectively, generating two capsule-deficient strains KP_1.6366 Δ*wzi* (785 nucleotides from 313 bp to 1097 bp were deleted from the *wzi* full sequence of 1434 bp) and KP_1.6366 Δ*wzy* (765 nucleotides from 131 bp to 895 bp were deleted from the *wzy* full sequence of 1170 bp) (Figure 2a,b). Then, the wild-type KP_1.6366 strain and two mutated strains, Δ*wzi* and Δ*wzy*, were cultivated in parallel under the same condition before cell collection. To harvest cells, we centrifuged each culture broth at a speed of 12,000× *g* for 15 min. As shown in Figure 2c, cell pellets of Δ*wzy* could be easily separated from the broth after centrifugation, and the supernatant was clearer than those of the wild-type and Δ*wzi* strains. With these three kinds of cell pellets, we next sought to use them to prepare cell extracts for setting up CFPS reactions. Since both *K. pneumoniae* and *E. coli* belong to the *Enterobacteriaceae* family, we initially tried to adopt the protocol used for *E. coli* CFPS systems [36,37]. After preparing cell extracts, we carried out CFPS reactions to synthesize sfGFP at 30 °C for 8 h. The results suggested that cell extracts prepared from the wild-type and Δ*wzi* strains showed very low protein synthesis activity. Clearly, the Δ*wzy* cell extract was highly active, which is capable of synthesizing about 50 μg/mL of sfGFP (Figure 2d). Therefore, on the basis of in vitro protein productivity, the Δ*wzy* strain was selected to develop and optimize our *K. pneumoniae* CFPS system in the following experiments.

### 2.2. Optimization of Cell Extract Preparation

**Figure 3:**
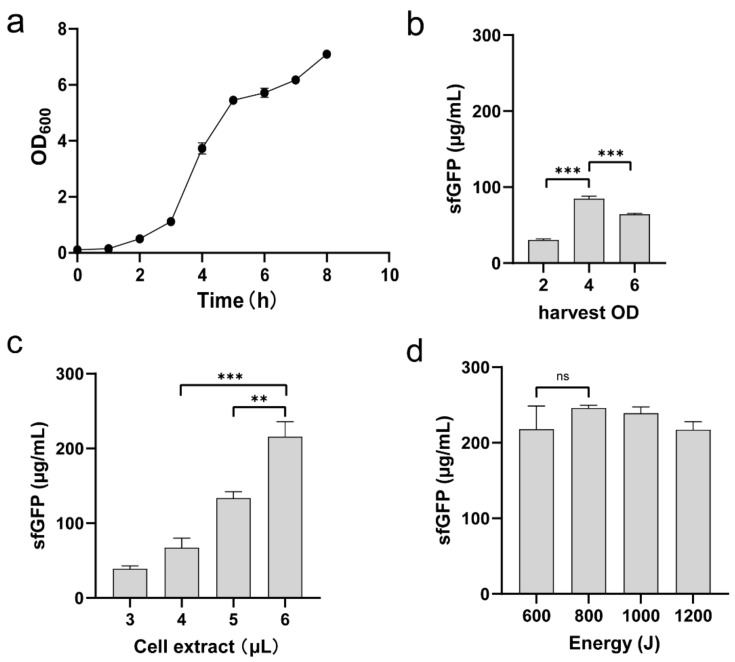
Optimization of cell extract preparation. (**a**) A representative cell growth curve of the strain KP_1.6366 Δ*wzy*. (**b**) Comparison of sfGFP yields with Δ*wzy* cell biomass harvested at different optical densities (OD_600_). (**c**) Evaluation of cell extract volume on cell-free synthesis of sfGFP. (**d**) Effect of sonication energy on the activity of cell extracts. Values show means with error bars representing standard deviations (s.d.) of at least 3 independent experiments. Student’s *t*-tests were used for statistical analysis, and *p* < 0.05 indicated statistical significance (** *p* < 0.01 and *** *p* < 0.001; ns, *p* > 0.05).

Having constructed a suitable capsule-deficient strain KP_1.6366 Δ*wzy*, we next set out to optimize the procedure of cell extract preparation. Cell extracts contain necessary elements, for example, ribosomes, aminoacyl-tRNA synthetases, protein translation-related factors, and chaperones, which are essential for transcription, translation, and protein folding [1,2]. Hence, it is crucial to prepare highly active cell extracts to support CFPS reactions. We focused our investigation on two key steps relative to extract preparation. First, we hypothesized that cell harvest conditions required to be assessed. In general, cells used for preparing cell extracts are harvested during the exponential growth phase, where cells are believed to divide rapidly and have high concentrations of active translation components. To determine the optimal time point for cell collection, we grew Δ*wzy* cells to different OD_600_ values from 2 to 6, covering a range of early to late exponential growth phase (see Figure 3a for a full cell growth curve). Then, cell extracts were prepared from each of these cultivations, which were used for cell-free synthesis of sfGFP to compare their activity. The results indicated that cell extracts prepared from the mid-exponential growth phase with an OD_600_ of 4 synthesized the highest yield of sfGFP at 84.8 ± 3.3 μg/mL (Figure 3b). This is in agreement with several other CFPS systems derived from *E. coli*, *Streptomyces*, and *Pseudomonas putida* [15,21,36]. Based on our data, we noticed that the productivity of different cell extracts varied, which is likely related to the composition and/or concentration of intracellular key elements for protein synthesis. To test this hypothesis, we added different amounts of cell lysates per 15 μL CFPS reaction, ranging from 3 (20%, *v*/*v*) to 6 μL (40%, *v*/*v*). Note that 4 μL of cell extract was used as a control. By doing this, we found that sfGFP yields were notably enhanced by adding more cell lysates, and the highest yield reached 215 ± 20.12 μg/mL with 6 μL of cell extract, a >2.5-fold improvement compared to the control reaction (Figure 3c). The increase of sfGFP yields indeed suggests that the concentration of translation-related elements in the CFPS reaction is a key factor for in vitro protein production.

Next, we sought to investigate the effect of total energy input during sonication on cell lysis and the resultant crude extract performance (i.e., protein synthesis ability). To do this, cell suspensions (1.4 mL per 1.5 mL Eppendorf tube) were lysed using different sonication energy inputs. Afterward, CFPS reactions were performed to determine the activity of each cell lysate. Our data indicated that the lysate generated with an input energy of 800 J led to a slightly higher sfGFP yield of 246 ± 3.58 μg/mL as compared to the other energy inputs (Figure 3d). However, it should be noted that once the volume of cell suspension changed, the sonication energy needs to be optimized accordingly to obtain the best condition for cell disruption. In principle, a low energy input would not be enough to disrupt all cells; on the other hand, a high level of sonication energy can sufficiently lyse cells but can also damage the activity of cell extracts due to heating introduced by many cycles of sonication.

### 2.3. Optimization of Reaction Conditions in K. pneumoniae CFPS

**Figure 4:**
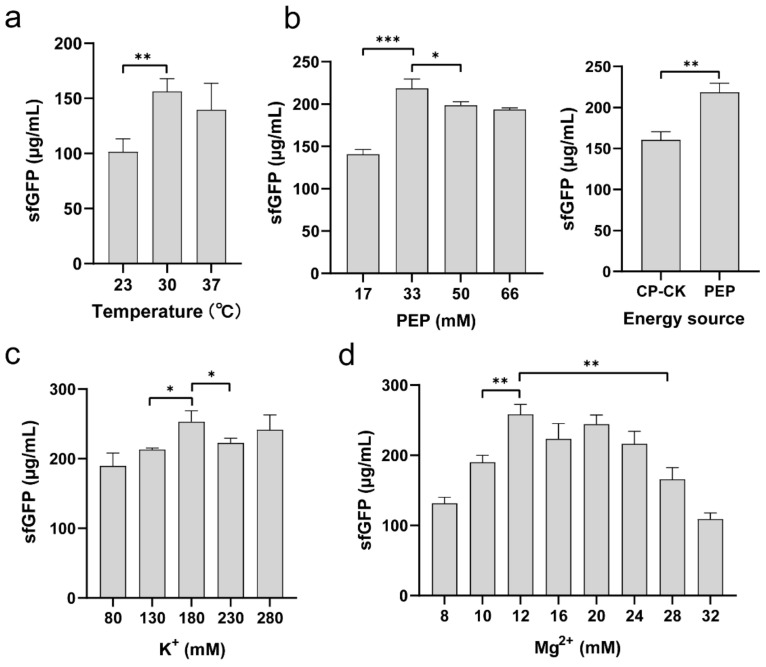
Optimization of CFPS reaction conditions. (**a**) Effect of reaction temperature on the sfGFP yield. (**b**) Effect of PEP concentration (**left**) and different energy regeneration systems (CP/CK and PEP, **right**) on cell-free synthesis of sfGFP. Optimization of (**c**) K^+^ and (**d**) Mg^2+^ concentrations in *K. pneumoniae* CFPS reactions. Values show means with error bars representing standard deviations (s.d.) of at least 3 independent experiments. Student’s *t*-tests were used for statistical analysis, and *p* < 0.05 indicated statistical significance (* *p* < 0.05, ** *p* < 0.01, and *** *p* < 0.001).

To further improve the productivity of the *K. pneumoniae* CFPS system, we next sought to optimize several key physicochemical parameters that are known to impact the protein yield. We started with the cell-free reaction temperature, because it affects protein synthesis rate, yield, and folding [15,21,36]. We ran cell-free reactions at three different temperatures of 23, 30, and 37 °C, respectively. The results showed that the medium temperature of 30 °C favored protein synthesis, which can increase the sfGFP yield by 54% and 12% higher than that of 23 and 37 °C, respectively (Figure 4a). This optimal reaction temperature is the same as some other CFPS systems, for example, *Bacillus subtilis*, *Vibrio natriegens*, and *Pichia pastoris* [20,22,38].

In CFPS reactions, PEP is often used as an energy substrate to generate ATP for protein translation. Thus, the amount of PEP supplied to CFPS might affect the efficiency of protein synthesis, which needs to be optimized for each cell-free system. To test this, we added different concentrations of PEP to the *K. pneumoniae* CFPS reactions. As shown in Figure 4b (left), the optimal concentration of PEP was found to be 33 mM, yielding 218.7 ± 11.02 μg/mL of sfGFP. A higher amount of PEP in CFPS reactions did not further increase but slightly reduce the protein yields. Moreover, we compared PEP with another energy regeneration system, i.e., creatine phosphate/creatine kinase (CP/CK), which is commonly used in eukaryotic organism-based CFPS systems [14], to see their effect on the protein production. The data showed that the sfGFP yield with PEP system was 1.36-fold higher than that of the CP/CK system (Figure 4b, right). As a result, PEP is a more suitable energy source for the *K. pneumoniae*-based CFPS system.

Finally, we set out to explore the impact of two significant cations (K^+^ and Mg^2+^) on the CFPS productivity, which have been shown previously to be critical ions for CFPS reactions [39,40]. We first investigated a range of K^+^ concentrations from 80 to 280 mM in the cell-free reaction mixture. We observed that the CFPS productivity increased steadily with increasing K^+^ concentration and the protein yield reached maximally at 180 mM of K^+^ ion (Figure 4c). This concentration is higher than that used in the *E. coli*-based CFPS system (130 mM K^+^). Subsequently, we varied the concentration of Mg^2+^ ion in the CFPS reactions. The data suggested that the highest sfGFP yield of 253 ± 15.79 μg/mL was achieved when the reaction supplemented with 12 mM of Mg^2+^ (Figure 4d), which is the same Mg^2+^ concentration used in the *E. coli* CFPS system. Overall, our optimized *K. pneumoniae* CFPS system is able to synthesize 5-fold more protein than that of the initial non-optimized reaction.

## 3. Materials and Methods

### 3.1. Bacterial Strains, Culture Media, and Plasmids

An industrial *K. pneumoniae* KP_1.6366 wild-type strain was used in this work [35]. Two capsule-deficient strains, Δ*wzi* and Δ*wzy*, were constructed as described below in Section 3.2. Pre-cultivation of *K. pneumoniae* cells was performed in LB medium, containing 10 g/L tryptone, 5 g/L yeast extract, and 10 g/L NaCl (pH 7.0). The 2× YTPG medium (10 g/L yeast extract, 16 g/L tryptone, 5 g/L NaCl, 7 g/L K_2_HPO_4_, 3 g/L KH_2_PO_4_, and 18 g/L glucose, pH 7.2) was used to grow *K. pneumoniae* for the preparation of cell extracts. All plasmids used in this work are listed in Table 1.

### 3.2. Genome Engineering of the Wild-Type K. pneumoniae KP_1.6366 Strain

To delete the capsule-associated genes from the genome of wild-type *K. pneumoniae* KP_1.6366, a CRISPR-Cas9/lambda-Red method was used as previously described [35]. Specifically, the two genes *wzi* and *wzy* were individually deleted from the wild-type strain, generating two engineered strains, KP_1.6366 Δ*wzi* and KP_1.6366 Δ*wzy*, respectively. In brief, the pCasKP-apr plasmid was electroporated into the wild-type KP_1.6366 strain to generate the pCasKP-apr-harboring strain. Then, the strain was inoculated into LB medium with 30 μg/mL apramycin and incubated at 30 °C. When the cell density reached an OD_600_ of ~0.2, a final concentration of 0.2% L-arabinose was added to induce the lambda-Red recombineering operon of the pCasKP-apr plasmid. After 2 h induction, the cells were harvested and prepared as electrocompetent cells by washing twice in sterile ice-cold 10% glycerol, and then concentrated approximately 500-fold. Then, 2 μL of the spacer-inserted pSGKP-km plasmid and 3 μL of 100 μM corresponding ssDNA donor complex were added to 50 μL of the electrocompetent cells. The cell-plasmid-ssDNA suspensions were transferred to prechilled 2 mm electroporation cuvette and electroporated at 2.5 kV, 200 Ω, and 25 μF. The pulsed cells were recovered in 1 mL of antibiotic-free medium for 1.5 h at 30 °C. Then, the incubated cells were plated onto the LB agar plates supplemented with 30 μg/mL apramycin and 50 μg/mL kanamycin. The desired gene deletion mutants were verified by colony PCR and Sanger DNA sequencing. After deletion of the *wzi* and *wzy* genes, both pCasKP-apr and pSGKP-km plasmids were cured successfully by cultivating cells in the LB medium supplemented with 5% sucrose at 37 °C. The spacer sequences for deleting the *wzi* and *wzy* genes in this study were designed using the sgRNAcas9 software [41].

### 3.3. Preparation of Cell Extracts

Different *K. pneumoniae* strains including wild-type, Δ*wzi*, and Δ*wzy* strains were pre-cultured in the LB medium overnight, respectively, at 34 °C and 250 rpm. Next, the overnight culture was used to inoculate 500 mL of 2× YTPG medium with an initial OD_600_ of 0.05. Then, the cells were cultivated at 34 °C and 250 rpm until the OD_600_ reached around 3. Afterward, the cells were harvested by centrifugation at 4 °C and 12,000× *g* for 15 min. The collected cells were washed with cold S30 buffer (10 mM Tris-acetate pH 8.2, 14 mM magnesium acetate, 60 mM potassium acetate, and 2 mM dithiothreitol) three times. After the final wash, the pelleted cells were resuspended in S30 buffer (1 mL/g of wet cell mass). Cell disruption was carried out with a Q125 Sonicator (Qsonica, Newtown, USA) at 50% of amplitude with a 10 s on/off lysis cycle for a total input energy of ~680 J. For each sonication, the volume of resuspended cells was 1.4 mL per 1.5 mL Eppendorf tube. Note that this cell extract preparation procedure follows a previous protocol optimized for *E. coli* [36], but it seems to work equally well here. After sonication, the lysate was centrifuged at 12,000× *g* and 4 °C for 30 min. The resulting supernatant as cell extract was collected, aliquoted, flash frozen in liquid nitrogen, and finally stored at −80 °C until further use.

### 3.4. Cell-Free Protein Synthesis (CFPS) Reactions

CFPS reactions (a total volume of 15 μL reaction mixture per 1.5 mL Eppendorf tube) were carried out at 30 °C for 8 h unless otherwise noted. Each reaction mixture contained the following reagents: 12 mM magnesium glutamate; 10 mM ammonium glutamate; 130 mM potassium glutamate; 1.2 mM ATP; 0.85 mM each of GTP, UTP, and CTP; 34 μg/mL folinic acid; 170 μg/mL of tRNA mixture (Roche, product no. 10109541001); 2 mM each of 20 standard amino acids; 0.33 mM nicotinamide adenine dinucleotide (NAD); 0.27 mM coenzyme A (CoA); 1.5 mM spermidine; 1 mM putrescine; 4 mM sodium oxalate; 33 mM phosphoenolpyruvate (PEP); 13.3 μg/mL plasmid; 100 μg/mL T7 RNA polymerase; and 27% (*v*/*v*) of cell extract. Note that all chemical reagents were dissolved in nuclease-free water (Invitrogen^TM^, ThermoFisher Scientific, Waltham, MA, USA), respectively, and their pH values were adjusted to 7.0–7.2 accordingly. The concentration of cell-free synthesized sfGFP was determined as described below to measure and optimize the productivity of the *K. pneumoniae*-based CFPS system.

### 3.5. Protein Quantification

After CFPS reactions, 2 μL of the reaction sample was mixed with 48 μL nuclease-free water in a 96-well plate with flat bottom. Then, measurements of the sfGFP fluorescence were performed using a BioTek SYNETGY H1 plate reader with excitation and emission wavelength at 485 and 528 nm, respectively. Afterward, the values of sfGFP fluorescence were converted to concentration (μg/mL) according to a linear standard curve made in house using purified sfGFP (Equation: y = 126.36x − 12972, *R*^2^ = 0.99, where y is the fluorescence, and x is the sfGFP concentration). Note that the lysate autofluorescence in the CFPS reaction without plasmids has been subtracted from all samples. For each protein quantification, at least three independent experiments were carried out using the same cell extract. Then, the protein concentration of each independent reaction was technically measured in triplicate.

## 4. Conclusions

In this study, we describe the development of a novel CFPS platform, which is derived from a non-model industrial bacterium *K. pneumonia*. To facilitate cell collection and disruption, we initially deleted the capsule formation-associated genes with the help of a CRISPR-Cas9/lambda-Red genetic tool. Using the capsule-deficient strain, we systematically optimized the key steps of cell extract preparation and significant physicochemical parameters for cell-free reactions. The final, optimized *K. pneumonia* CFPS system enabled a high-yield production of sfGFP with a maximum value of more than 250 μg/mL. A summary of the newly developed cell-free system is shown in Table 2. Such high productivity will make *K. pneumonia* cell-free system not only a valuable addition to the current CFPS toolkit, but also a promising platform for broad applications in the field of biotechnology and synthetic biology. In particular, the system holds great potential to construct in vitro metabolic pathways for the synthesis of 2,3-butanediol, gluconic acid, and 1,3-propanediol to avoid the issues of in vivo production including broth viscosity, by-product formation, and even pathogenicity [27,28,30]. Moreover, we envision that the high-yield *K. pneumonia* CFPS system together with many other cell-free platforms will be effectively used to produce value-added macromolecules like therapeutic proteins as well as small molecules such as agricultural and industrial chemicals.

## Figures and Tables

**Table 1:** Plasmids used in this study.

Plasmid	Description	Source
pCasKP-apr	Expression of Cas9 and λ-Red proteins in *K. pneumoniae*	Addgene # 117231
pSGKP-km	Expression of sgRNA in *K. pneumonia*	Addgene # 117233
pSGKP-km_*wzi*	pSGKP-km derivative with *wzi* spacer	This study
pSGKP-km_*wzy*	pSGKP-km derivative with *wzy* spacer	This study
pBECKP-km	Expression of APOBEC1-nCas9 fusion protein and sgRNA in *K. pneumonia*	Addgene # 117235
pJL1-sfGFP	Expression of the reporter protein sfGFP in CFPS	Addgene # 69496

**Table 2:** The optimized *K. pneumonia* CFPS system.

Key Parameters	Description
Cell extract preparation	
Strain	*K. pneumonia* Δ*wzy*, a capsule-deficient strain
Cultivation	500 mL of 2× YTPG, 34 °C, 250 rpm
Collection	OD_600_ = 4
Lysis	Sonication, input energy: 800 J
CFPS reaction	
Total volume	15 μL in 1.5 mL Eppendorf tube
Cell extract	6 μL per reaction (40%, *v*/*v*)
Energy	PEP, 33 mM
Mg^2+^ ion	12 mM
K^+^ ion	180 mM
Temperature	30 °C
Other components	See “Section 3.4.”

## Data Availability

Not applicable.

## Sample Availability

Samples are not available from the authors.
